# Neuroendocrine Effects of Short-Bout Aerobic Exercises in Individuals With Alcohol Use Disorder: A Quasi-experimental Study

**DOI:** 10.7759/cureus.78921

**Published:** 2025-02-12

**Authors:** Rahul Shaik, V R Abhinaya Ravada, Prasad Yeddu, Danturty L Lalitha

**Affiliations:** 1 Physiotherapy in Neurology, Great Eastern Medical School and Hospital (GEMS) College of Physiotherapy, Srikakulam, IND; 2 Psychiatry, Great Eastern Medical School and Hospital (GEMS), Srikakulam, IND; 3 Physiotherapy, Great Eastern Medical School and Hospital (GEMS), Srikakulam, IND; 4 Biochemistry, Great Eastern Medical School and Hospital (GEMS), Srikakulam, IND

**Keywords:** alcohol craving, alcohol use disorder (aud), de-addiction, short-bout aerobic exercises, withdrawal anxiety

## Abstract

Background

Half of the chronic alcoholic population usually relapses after extensive treatment in de-addiction centers. This craving is due to the reduced release of hormones in the hypothalamic-pituitary-adrenal, hypothalamic-pituitary-gonadal, and hypothalamic-pituitary-thyroid axes. Exercise has a positive influence on some of these neuroendocrine axes, releasing corticotrophin, cortisol, oxytocin, and vasopressin. Thus, there is a need to study the additional benefits of exercises during de-addiction programs on craving and anxiety in acute alcohol-de-addicted individuals. This study aims to compare blood levels of oxytocin, cortisol, and vasopressin and relate these factors’ influence on craving and anxiety in individuals performing short-bout aerobics regularly and those not performing any type of exercise in an alcohol de-addiction center.

Methodology

In this quasi-experimental study, 76 individuals from an alcohol de-addiction center were selected and divided into two equal groups using systematic random sampling after obtaining ethical clearance from the institutional ethical committee. The experimental group was treated with two sessions of aerobic exercises each day for five days a week. One session consisted of five repetitions of cycling, and the other session consisted of five repetitions of fast walking on a computerized treadmill. In both sessions, two minutes of workout were followed by three minutes of rest. The control group was not given any exercise protocol but was treated with de-addiction medications and advised regular counseling with a psychiatrist similar to the experimental group. The treatment was given for one month. Blood levels of oxytocin, cortisol, and vasopressin were collected before and after intervention in both groups. Anxiety levels and alcohol cravings were measured in both groups using the Hamilton Anxiety Scale and Penn Alcohol Craving Scale, respectively, before starting the treatment and after one month of treatment. Based on the normality test status, non-parametric statistical tests were used for analyzing data related to oxytocin, cortisol, and vasopressin levels. Anxiety and craving levels were analyzed using parametric statistical tests.

Results

An extremely significant difference was noted (p < 0.001) before and after treatment in both groups. Individuals in the experimental group treated with short-bout exercises significantly improved their blood levels of oxytocin, cortisol, and vasopressin when compared to the control group. The same levels of improvement were observed for craving and anxiety when compared to the control group.

Conclusions

Short-bout aerobic exercises showed better results in treating alcohol craving and anxiety in individuals with alcohol use disorder admitted to a de-addiction center. These positive effects may be related to the influence of short-bout aerobic exercises on neuroendocrine effects, which regulate the blood levels of oxytocin, cortisol, and vasopressin.

## Introduction

Alcohol use disorder (AUD) is a substance use disorder identified as a major factor contributing to illness, disability, and mortality. According to the International Classification of Diseases, Tenth Edition, alcohol is one of the risks/causes for more than 30 conditions. Heavy alcohol consumption refers to more than 40 and 20 g of pure alcohol intake per day for men and women, respectively [1]. AUD is the third-most disabling disease category in high-income countries and the fourth-most disabling disease category in low- to middle-income countries. Alcohol causes approximately 3.3 million deaths every year globally and contributes to 5.1% of the global burden of alcohol-related diseases. Negative effects on people’s mental and physical aspects have been observed from uncontrolled and excessive alcohol consumption [2]. Alcohol-related disorders are the most common disorders in diverse countries, with a worldwide contribution of 4.5% to all diseases and injuries [3]. Alcohol abuse has been a significant cause of death [4]. In 2005, alcohol dependents made up 17% of the 62 million estimated alcohol users [5]. In India, the prevalence of AUD among adult men was 4.5% in 2010, 10% in 2014, and 9% in 2016 [6].

Psychiatrists in de-addiction centers typically use medications and behavioral therapy to reduce alcohol dependency and cravings. Anxiety, tremors, nausea, insomnia, and, in severe cases, seizures have been observed after withdrawal. Although up to half of the individuals with AUD show withdrawal symptoms after stopping drinking, only a small percentage require medical treatment for detoxification. Some individuals may even be able to reduce their drinking. Alcohol intake is known to modulate plasma concentrations of neuroendocrine peptides. However, recent study findings suggest that the endocrine system may not only respond passively to alcohol intake but also actively modulate alcohol intake behavior [7]. The most coherent body of data concerns the hypothalamic-pituitary-adrenal (HPA) cortisol axis, with low corticotrophin-releasing hormones associated with more intense cravings and increased probability of relapse after acute detoxification [8]. Although most of the currently available data demonstrate association rather than causality between neuroendocrine changes and alcohol-related behaviors, they provide testable hypotheses and open up perspectives on the neuroendocrine axis. These hypotheses and perspectives can be explored to obtain better results for alcohol dependency.

The HPA axis has three components, namely, the hypothalamus, anterior pituitary, and adrenal cortex, which are structurally independent. These structures intimately interact through the release of neuroendocrine messengers and the activation or inhibition of the nervous system, influencing the functions of most of the body’s organs and tissues [9]. Such interactions include functions of peptide hormones, corticotrophin-releasing factor, adrenocorticotropic hormone, and arginine vasopressin and their specific receptors, as well as smaller molecular species such as corticosteroids [10]. The HPA axis can be stimulated physiologically with exercises [11]. The minimum intensity of exercise required to produce a cortisol response is 60%. A few studies have also noted changes in HPA with 40% of VO_2_ max after 90 minutes of exercise [12].

With a relapse rate ranging between 60% and 90%, AUD is considered one of the most significant problems in alcoholic patients [13]. Various treatments have been tried to reduce it. Marlatt and Witkiewitz [14] emphasized a treatment approach with lifestyle modifications to prevent relapse behaviors in their social learning model of the relapse process in addictive disorders. The positive effects of physical exercises have not been investigated sufficiently in terms of reduced relapse rates after alcohol withdrawal. In fact, among the strategies to prevent relapse, exercise offers several advantages [15-17]. These advantages, in comparison to those of pharmacological treatment, include improvement in health and feelings of wellness, cost-effectiveness, flexibility, accessibility, and minimal side effects [18].

Few studies have confirmed that, in alcohol-dependent patients, pleasure ratings after performing exercises are higher when compared to drinking alcohol [19]. Furthermore, Zourbanos et al. theoretically suggested the use of exercise as a strategy to increase β-endorphin levels and control the urge to consume alcohol [20]. Knowledge about the effects of exercise on alcohol urges and the physiological factors controlling alcoholic patients’ cravings is scarce. Therefore, this study aims to examine the effects of acute aerobic exercises of moderate intensity on the neurophysiological basis of controlling alcohol urges and anxiety in chronic alcoholics participating in a rehabilitation program. There is a need to determine the influence of short-bout aerobic exercises on oxytocin, cortisol, and vasopressin to provide a possible mechanism explaining the control of cravings and anxiety during de-addiction therapy.

## Materials and methods

**Figure 1:**
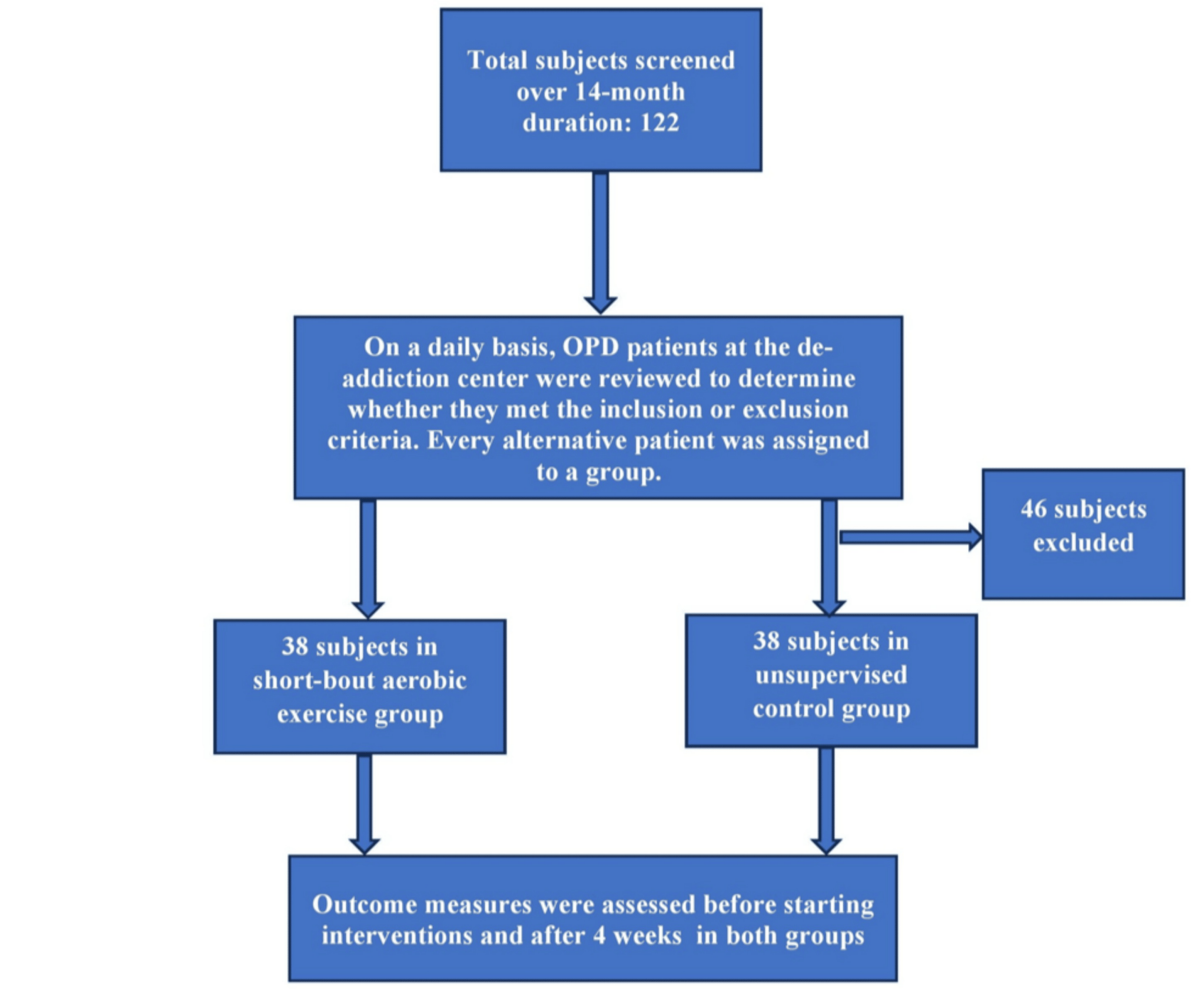
Sampling and sample allocation flowchart.

In this quasi-experimental design study, individuals with mild-to-moderate alcohol addiction were selected from a de-addiction center at the Department of Psychiatry, Great Eastern Medical School and Hospital, Ragolu, Srikakulam, India. The individuals were randomly divided into two groups after obtaining institutional ethical committee clearance. The research procedure was explained to the individuals and their attendants in detail, and their informed consent was obtained. The inclusion criteria were patients dealing with alcohol addiction for more than one year who were admitted to the de-addiction center. All subjects were men between the ages of 30 and 50 years. The exclusion criteria were alcohol addicts with severely impaired cognition, psychologically unstable individuals, and known diabetics. This research was a part of a faculty research grant and the total duration allotted was two years. To meet the time constraints, the suitable subjects were screened over a period of 14 months. Each subject was screened to determine whether they met the inclusion and exclusion criteria, and every alternative patient visiting our outpatient department was allocated to each group. A total of 76 subjects were enrolled in the study who were equally assigned to the short-bout exercise group and control group using the systematic random sampling technique (Figure 1).

**Figure 2:**
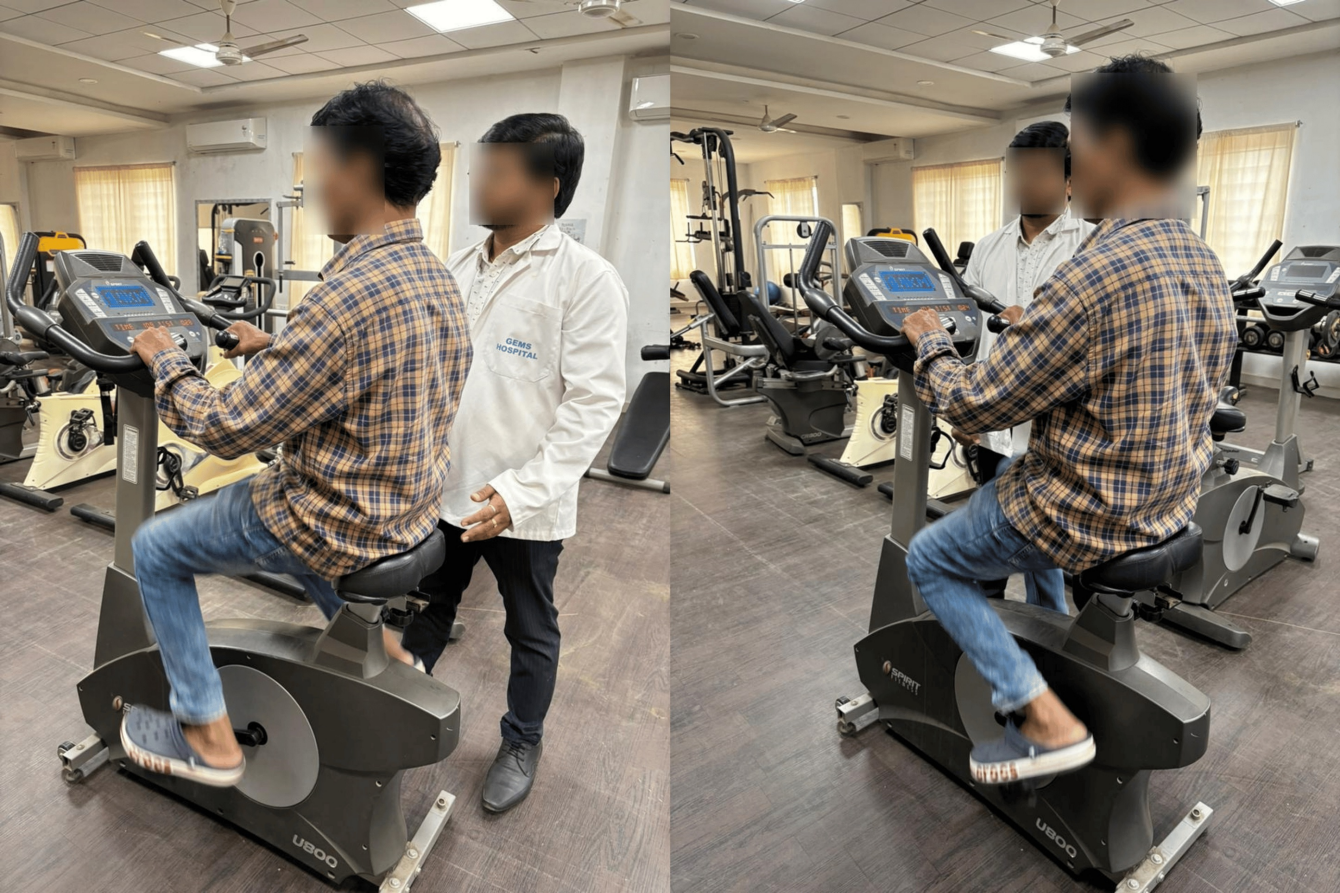
A subject performing short-bout aerobic exercises on a static cycle.

All subjects in the experimental group were treated with short-bout aerobic exercises using a static bicycle and computerized treadmill. Before starting an exercise regimen, the subject’s rate of perceived exertion was measured and the exercise intensity was designed to match their comfort level. The maximum exercise duration in each bout was two minutes. A rest period of three minutes was allowed between subsequent exercises. The exercises in the first session included two minutes of fast cycling with three minutes of rest repeated five times. Then, a 10-minute break was provided to allow subjects to cool down and warm up for the next exercise session (Figure 2).

**Figure 3:**
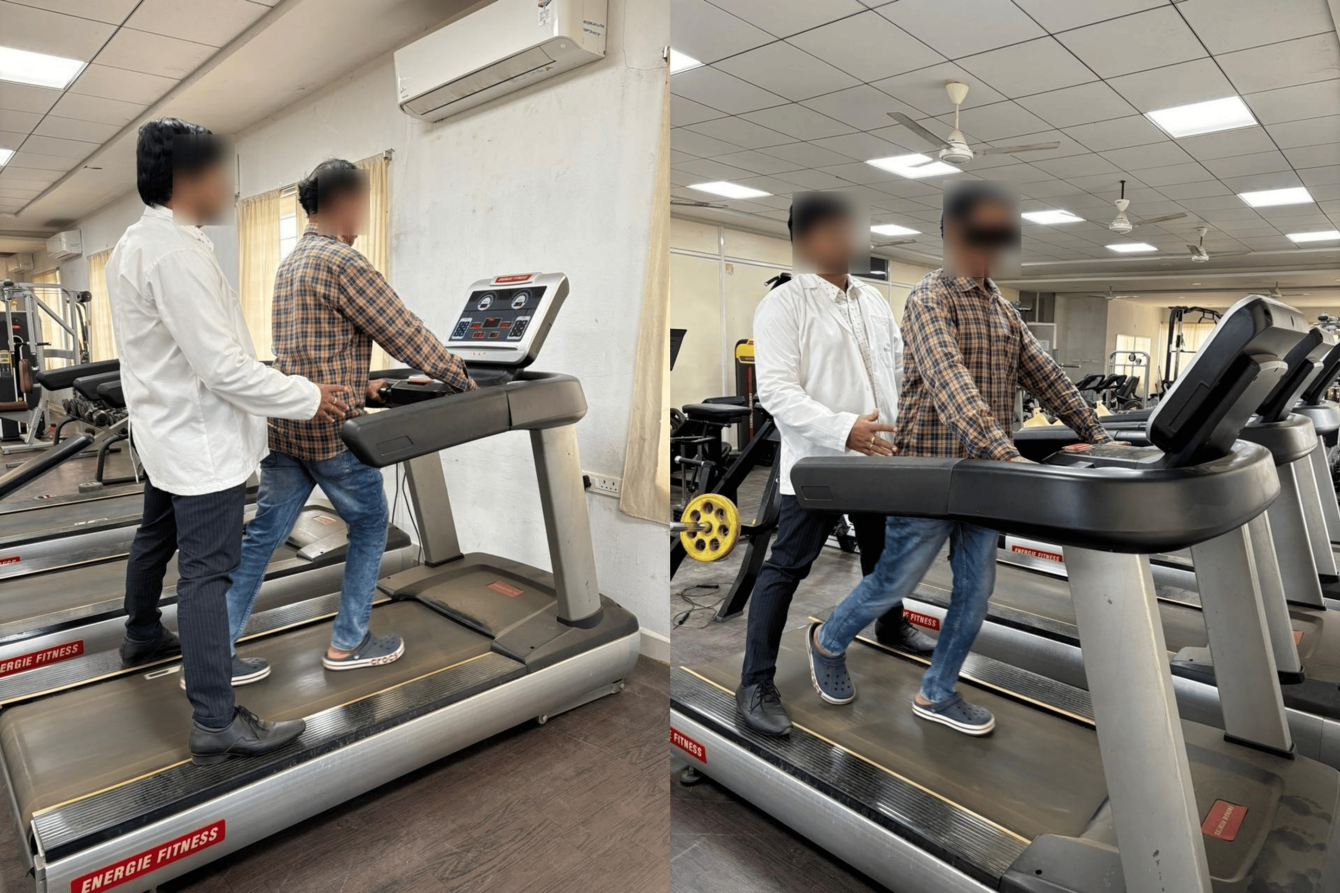
A subject performing short-bout aerobic exercises on a treadmill.

In the second short-bout aerobic exercise session, subjects were asked to walk fast on a computerized treadmill without inclination for two minutes. They were allowed to rest for three minutes between each bout. This exercise was repeated five times (Figure 3). All exercises were conducted five days a week for one month under the physiotherapist’s supervision.

The control group was not given any exercise protocol but was treated with de-addiction medications and regular counseling with a psychiatrist similar to the experimental group. The treatment was given for one month for subjects in both groups. Blood levels of oxytocin, cortisol, and vasopressin were collected around 8 am before the start of the intervention and the next day after the completion of the last session of the exercise (after the completion of the intervention duration) from the subjects in both groups. Anxiety levels and alcohol cravings were measured in both groups using the Hamilton Anxiety Scale and Penn Alcohol Craving Scale, respectively, before starting the treatment and after one month of treatment. The evaluators were blinded to the research objectives and interventions.

Data analysis

Statistical analysis was performed using SPSS software (IBM Corp., Armonk, NY, USA) and MS Excel 2010 (Microsoft Corp., Redmond, WA, USA). All descriptive statistical data were presented in the form of means and standard deviations. The data were also tabulated and graphically represented. The Kolmogorov-Smirnov test was used to examine assumptions about the data sampled from Gaussian distributions. Data on blood levels of oxytocin, cortisol, and vasopressin failed the normality test. Thus, the Wilcoxon matched-pair signed-rank test was used for within-group comparisons, and the Mann-Whitney U test was used for between-group comparisons for the above outcome measures. The paired t-test was used to compare the means of pre- and post-intervention values of the Hamilton Anxiety Scale and Penn Alcohol Craving Scale. The independent Student’s t-test (unpaired-t test) was conducted to compare the means of two post-intervention values of the Hamilton Anxiety Scale and Penn Alcohol Craving Scale. P-values <0.05 were considered statistically significant.

## Results

Subjects in both groups were at the same level of addiction. There was no significant difference in blood levels of oxytocin, cortisol, vasopressin, craving, and anxiety in both groups before starting the interventions (Table 1).

**Table 1:** Baseline characteristics of subjects in both groups.

Patient characteristics	Experimental group	Control group	P-value
Age	43.3	44.8	0.199
Weight	65.1	64.9	0.815
Body mass index	20.1	20.1	0.891
History of alcoholism in years	10	10.9	0.116
Number of attempts at withdrawal	0.8	0.8	0.892
Oxytocin	15.2 ± 2.2	15.2 ± 1.9	0.867
Cortisol	35.9 ± 2.9	35.8 ± 2.5	0.867
Vasopressin	13.6 ± 2.0	14.5 ± 2.0	0.406
Hamilton Anxiety Scale	31 ± 2.5	30.7 ± 2.5	0.615
Penn Alcohol Craving Scale	24.9 ± 1.6	24.7 ± 1.4	0.582

Extremely significant differences were noted (p < 0.0001) before and after treatment in both groups. Subjects treated with short-bout exercises greatly improved their blood levels of oxytocin, cortisol, and vasopressin compared to the control group. Similarly noteworthy levels of improvement were observed for craving and anxiety (Table 2).

**Table 2:** Comparison of post-intervention values of outcome measures in both groups.

Outcome measures	Short-bout aerobic exercise group			Control group			Z value	P-value
	Mean	SD	SE	Mean	SD	SE		
Oxytocin	39.6	±5.346	0.867	29.6	±4.084	0.662	19.967	<0.0001
Cortisol	15.3	±2.344	0.380	27.2	±1.567	0.254	-37.320	<0.0001
Vasopressin	2.8	±0.843	0.136	6.2	±1.018	0.165	-15.458	<0.0001
Hamilton Anxiety Scale	20.1	±1.679	0.272	23.2	±1.658	0.269	-10.302	<0.0001
Penn Alcohol Craving Scale	16.0	±2.388	0.387	19.6	±1.516	0.246	-11.150	<0.0001

## Discussion

This study obtained positive results for alcohol craving and anxiety in subjects with AUD in a de-addiction center who performed regular short-bout aerobic exercises. Improvements in blood levels of oxytocin, cortisol, and vasopressin were evident. Even though there is a wide variation in the effects of exercises among alcoholics in the existing literature for the individual factors studied in this research, the results of the outcome measures in general support the neurophysiological basis of the effects of aerobic exercises on reducing craving and anxiety during the acute phase of alcohol withdrawal.

Exercise is associated with a euphoric feeling during and after a session [21,22]. Although this study did not consider β-endorphin levels as one of the outcomes, exercise-induced increments in β-endorphin could positively influence heavy drinkers’ mood and inhibit the desire for alcohol. Therefore, the influence of exercise on the endogenous opioid system can be considered an effective factor in alcohol abstinence and reduced alcohol cravings. The functions of the endogenous opioid system mainly involve modulation of the response to pain, reward and reinforcement, and regulation of functions such as thermoregulation and energy substrate mobilization [23-26].

During acute stress, HPA axis hyperactivity has been noted in animal studies, leading to increased cortisol production and other physiological responses. Oxytocin is considered one of the major factors regulating numerous social behaviors such as social reward, bonding, and aggression. It also regulates a few non-social behaviors such as stress and anxiety. Oxytocin plays a regulatory role in anxiety and stress, with studies showing its ability to lower cortisol levels and reduce subjective feelings of stress when administered during challenging social or stressful situations [27]. Oxytocin administration has been successfully linked to the reduction of psychopathological anxiety [28] and stress in late pregnancy in rats [29]. Similarly, the administration of oxytocin has been shown to reduce the intake and preference of alcohol, especially in rats [30] and mice [31]. In drug-addicted individuals, oxytocin can be considered a therapeutic target to improve mood and socio‐affiliative behaviors [32-34]. There was a 2.5-time increment in salivary oxytocin in both men and women after just 10 minutes of running [35]. Because of inconsistency in the validity of salivary oxytocin in yielding intra-individual variation [36], another study evaluated blood levels of oxytocin. Non-pharmacologically, aerobic exercises showed beneficial effects in improving blood oxytocin levels, causing stress reduction in pregnant women [37], improving sports performance [38], and improving sleep quality [39]. The results of this study also indicated promising increments in blood oxytocin levels after short-bout aerobic exercises.

Stephens and Wand concluded that stress, through its effect on the HPA axis and glucocorticoid release, plays a central role in the development and maintenance of alcohol dependence. Understanding these mechanisms could lead to better therapeutic approaches for treating individuals with AUDs, especially those who have significant stress-related components in their addiction [40]. People with heightened adrenal sensitivity may experience more pronounced alcohol cravings. In these individuals, the release of cortisol in response to stress may be amplified, making alcohol a more appealing coping mechanism due to its ability to temporarily alleviate heightened stress or anxiety. This dynamic could lead to a vicious cycle where stress increases cortisol, which increases alcohol craving, which, in turn, exacerbates stress and cortisol production. Hence, cortisol level in blood was used as the second biomarker in this study. Research has confirmed that measures to reduce adrenal sensitivity, stress, and craving also reduce instances of alcohol relapse after a few days of withdrawal [41]. Mücke et al. emphasized the need for experimental studies using longitudinal designs to determine the effects of physical activity on stress reactivity [42]. The results of this study support the existing notion that acute aerobic exercises such as incremental bicycle testing, endurance training, and relaxation programs significantly change cortisol levels [43].

During withdrawal, vasopressin levels usually increase as a response to the patient’s recovery from a chronic state of dehydration. This may cause fluid retention in the body [44]. Intense aerobic exercises increase blood vasopressin levels, helping regulate fluid balance and blood pressure during physical exertion. At the same time, moderate aerobic exercises reduce hypophyseal vasopressin [45]. A few studies disagree with the results of this study, particularly regarding the reduction of vasopressin levels through short-bout less intense aerobic exercises. These studies have found inconsistent evidence about the effects of exercise on vasopressin levels in AUDs [46,47]. The reduction in vasopressin levels compared to baseline values in this study may not be attributed purely to aerobic exercises. In individuals going through acute withdrawal, responses to exercises can vary depending on factors such as accommodation to exercise, exercise intensity [48], acute changes in the rennin-angiotensin-aldosterone system [49], vascular tone, blood pressure, sodium levels [50], and urine output. These factors may influence vasopressin levels. Hence, there is a need to study the detailed effects of exercises on vasopressin levels in AUDs, taking into account all these factors in further studies. Further, there is a need for randomized controlled studies and to control the potential biases due to group allocation techniques to confirm these changes.

## Conclusions

Individuals with AUD in a de-addiction center treated with short-bout exercises showed an increment in blood levels of oxytocin while a decrease in cortisol and vasopressin, along with a reduction in cravings and anxiety, after one month of treatment. These positive effects may be related to the influence of short-bout aerobic exercises on neuroendocrine effects.
